# Progress against childhood and adolescent acute lymphoblastic leukaemia in the Netherlands, 1990–2015

**DOI:** 10.1038/s41375-020-01024-0

**Published:** 2020-08-21

**Authors:** Ardine M. J. Reedijk, Jan Willem W. Coebergh, Hester A. de Groot-Kruseman, Inge M. van der Sluis, Leontien C. Kremer, Henrike E. Karim-Kos, Rob Pieters

**Affiliations:** 1 Princess Máxima Center for Pediatric Oncology, Utrecht, The Netherlands; 2 Department of Public Health, Erasmus University Medical Center, Rotterdam, The Netherlands; 3 Dutch Childhood Oncology Group, Utrecht, The Netherlands; 4 Department of Research, Netherlands Comprehensive Cancer Organisation (IKNL), Utrecht, The Netherlands

**Keywords:** Cancer epidemiology, Paediatrics

## Abstract

We assessed the epidemiologic progress against childhood and adolescent acute lymphoblastic leukaemia (ALL) in the Netherlands over a 26 year period. ALL patients <18 years were selected from the Netherlands Cancer Registry and the Dutch Childhood Oncology Group. Trend analyses were performed over time and by age group and ALL subtype. Between 1990 and 2015, 2997 ALL patients were diagnosed, i.e. 115 patients (range 87–147) per year. Overall incidence remained stable at 37 per million children, despite increases for B-cell precursor ALL (BCP-ALL) at age 10–14 years (AAPC + 1.4%, *p* = 0.04) and T-cell ALL at 15–17 years (AAPC + 3.7%, *p* = 0.01). Five-year survival increased from 80% in 1990–94 to 91% in 2010–15 (*p* < 0.01). Mortality decreased by 4% annually (*p* < 0.01). Patients 15–17 years were increasingly treated in a paediatric oncology centre, from 35% in 1990–94 to 87% in 2010–15 and experienced a 70% reduction of risk of death compared to those treated outside such a centre (*p* < 0.01). Significant progress against childhood ALL has been made in the Netherlands, visible by improved survival rates coinciding with declining mortality rates. These outcomes were accompanied by stable incidence rates, despite increases for BCP-ALL at age 10–14 years and T-cell ALL at age 15–17 years.

## Introduction

Increases in incidence of childhood acute lymphoblastic leukaemia (ALL) have been reported at the beginning of the 21st century [1–5]. No clear explanations for these increases could be given in the absence of specific causes. ALL is the most common cancer among children, as well as the most frequent cause of death from cancer below the age of twenty [6, 7]. Incidence and mortality trends are summary measures that provide snapshots of a long-term, time-dependent process [8]. Recent population-based studies for paediatric ALL focusing on incidence and mortality are limited in literature and are lacking for the Netherlands.

Since the early 1970s, treatment of children with ALL has been organised with national treatment protocols in the Netherlands. At that time the Dutch Childhood Leukemia Study Group (DCLSG, since 2002 extended to the Dutch Childhood Oncology Group [DCOG]) was established. The DCLSG/DCOG has a trial and data centre, with a reference diagnostic laboratory for leukaemias, and it also coordinates clinical trials, since 2003 also for solid tumours. Most recent changes in therapy were improvements in chemotherapy and better ways to stratify patients to receive less or more intensive therapy [9–12]. Trends in childhood ALL survival have been published in relation to therapeutic developments in several European countries, Japan and the US [13]. Both Pastore et al. [14] and Stiller et al. [15] have examined that changes in population-based survival parallel those reported by the relevant clinical trials. The increasing level of participation in trials, facilitated by the organisation of specialised care, has underpinned the substantial improvements in survival seen at the population level [15].

The overall aim of this study was to assess the progress made for children and young adolescents with ALL in the Netherlands since 1990 by analysing trends in incidence and survival against the background of subsequent treatment regimens. Data from the Netherlands Cancer Registry (NCR) were combined with detailed leukaemia and treatment characteristics from the DCOG registry. Mortality data on cause of death were derived from the website of Statistics Netherlands. In addition, detailed analyses were made regarding ALL subtype and site of treatment.

## Patients and methods

### Study population

Patients aged <18 years and diagnosed with ALL (ICD-O-3 M9811-9818 and M9835-9837) from January 1990 to December 2015 were extracted from the NCR. For completeness a linkage with DCOG was performed and after this linkage the ALL subtype, site of treatment and treatment protocol could be determined for patients known at the DCOG registry. A total of 2947 patients with ALL from the NCR were linked with 2882 patients from the DCOG, yielding 2997 patients eligible for inclusion (Supplementary Fig. 1). In case of discrepancies in morphology, DCOG data were preferred over NCR data because of their role as a reference laboratory. For patients ascertained in the NCR only, morphology codes (according to the International Classification of Diseases for Oncology (ICD-O)) as registered in the NCR were taken. ALL may be of B-cell precursor (BCP) or T-cell (T-cell) lineage [16]. For 11 patients (<1%) the subtype was unknown.

### The Netherlands Cancer Registry

The nationwide population-based NCR is maintained and hosted by the Netherlands Comprehensive Cancer Organisation (IKNL) and has a national coverage since 1989 with a completeness of at least 96% of all newly diagnosed malignancies in the Netherlands [17]. The NCR is notified by the Nationwide Network and Registry of Histopathology and Cytopathology, and the National Registry of Hospital Discharges. Retrospectively, data is extracted on patient, tumour and treatment characteristics. Primary therapy started within 9 months after diagnosis is recorded following order of administration and includes radiotherapy, systemic chemotherapy, and stem cell transplantation (SCT). Information on vital status (alive, dead, or emigration) is obtained by annual linkage of the NCR with the Nationwide Population Registries Network that holds vital statistics on all residents in the Netherlands. Last linkage was at February 1st 2019.

### Registry of the Dutch Childhood Oncology Group

The centrally reviewed results of bone marrow, peripheral blood and spinal fluid samples taken at diagnosis are registered at the DCOG database. ALL diagnosis is based on a combination of cytomorphology, immunophenotyping and –increasingly– (molecular) cytogenetics [12]. Baseline patient and leukaemia characteristics (e.g., sex, age, white blood cell count at diagnosis, pre-existing syndromes and cytogenetics) are collected from the treating hospitals and included in the database. Eligibility and inclusion in specific clinical trials or treatment protocols are centrally registered at the DCOG. For these “in-trial patients” details regarding diagnosis, treatment, response to treatment, toxicity and outcome including relapse(s), second malignancy, and death were also registered. Five consecutive ALL treatment protocols (ALL7 – ALL11) were active during our study period [9–12], plus specific protocols for infants, patients aged <1 year, since 1999 (Interfant) [18, 19] and for Philadelphia-chromosome-positive ALL (EsPhALL) since 2005 [20] (Supplementary Fig. 2). Only patients treated in the seven paediatric oncology centres in the Netherlands were included in the DCOG registry. In the 1990s treatment was also performed in some non-university hospitals, under supervision of one of the paediatric centres. For the site of treatment analyses patients were considered as being treated outside a paediatric oncology centre if they were unknown in the DCOG registry.

### Mortality data

Disease-specific mortality rates from 1980 to 2016 were derived from Statistics Netherlands (CBS). Because of privacy regulations, linkage between the NCR and CBS is not allowed in the Netherlands on a routine base. The lymphoid leukaemia (LL) specific ICD-9 code “204” and ICD-10 code “C91” were used to identify the number of persons who died from LL. Mortality data by age at death were presented by 5-year age groups (i.e., 0–4, 5–9, 10–14, and 15–19 years).

### Statistical analyses

Characteristics of the study population were described as percentages in relation to the following five periods of diagnosis: 1990–94, 1995–99, 2000–04, 2005–09, and 2010–15. Differences among categorical variables were tested with the χ2 tests.

Annual incidence and mortality rates were calculated per million person years, using the annual mid-year population size as obtained from Statistics Netherlands. Rates were age-standardised according to the age structure of the World standard population for age ranges 0–14 year, 0–17 year for estimation of incidence rates, and 0–19 year for mortality rates [21]. Linear regression modelling assessed trends over time (i.e. time period 1990–2015 for incidence and time period 1980–2016 for mortality). A regression line was fitted to the natural logarithm of the incidence and mortality rates, including calendar year as a continuous variable [21]. Results were reported as average annual percent changes (AAPC) along with the corresponding 95% confidence interval (CI) and *p* values.

Survival time was calculated as the time elapsed between the date of diagnosis and the date of death due to any cause (event) or date at last follow-up (i.e. alive or censored). Traditional actuarial survival analysis was used to calculate overall survival (OS) at 5 and 10 years after diagnosis. Changes over time in observed 5-and 10-year survival were evaluated with a p-trend analysis for period of diagnosis, sex, age at diagnosis, ALL subtype, and site of treatment by using parametric survival models (streg), adjusted for follow-up time (in years). To evaluate their effect on the risk of dying per period of diagnosis, these parameters were entered in a multivariable analysis model. For survival analyses according to treatment protocol, patients eligible and treated according to the protocol were included (DCOG patients only).

All analyses were performed with STATA/SE 14.1 (StataCorp LP, College Station, Texas, USA). Joinpoint regression program (version 4.5.0.1) was used to check for incidence trend transitions during the study period [22]. A *p* value < 0.05 was considered statistically significant.

### Role of the funding source

The funding source had no role in the study design, data collection, analyses and interpretation of the results, nor in writing of this manuscript.

## Results

### Patient and leukaemia characteristics

Between 1990 and 2015, 2997 children and adolescents aged <18 years were diagnosed with ALL in the Netherlands and analysed in this study. The majority of patients had a diagnosis confirmed by the reference laboratory of the DCOG (96%). Median age at diagnosis was 5 years (interquartile range 3–9 years). More boys than girls were diagnosed with ALL (male to female ratio (M:F ratio) being 1.4) (Table 1). Patients below five years were mainly diagnosed with BCP-ALL (94%), decreasing with age to 73% of the patients aged 15–17 years.

**Table 1:** Patient characteristics of patients aged < 18 years with acute lymphoblastic leukaemia in the Netherlands between 1990 and 2015.

	Total		Average per year	Period of diagnosis										*p-Chi2*
				1990–94		1995–99		2000–04		2005–09		2010–2015^c^		
	*N*	%	*N*	*N*	%	*N*	%	*N*	%	*N*	%	*N*	%	
	2997		115	481	16%	589	20%	640	21%	585	20%	702	23%	
Age groups (years)														0.04
0	90	3%	3	10	2%	16	3%	24	4%	22	4%	18	3%	
1–4	1385	46%	53	237	49%	292	50%	295	46%	244	42%	317	45%	
5–9	796	27%	31	129	27%	149	25%	166	26%	180	31%	172	25%	
10–14	479	16%	18	65	14%	89	15%	114	18%	84	14%	127	18%	
15–17	247	8%	10	40	8%	43	7%	41	6%	55	9%	68	10%	
Sex														0.02
Male	1744	58%	67	266	55%	373	63%	383	60%	325	56%	397	57%	
Female	1253	42%	48	215	45%	216	37%	257	40%	260	44%	305	43%	
Site of treatment														<0.01
Paediatric oncology centre	2882	96%	111	452	94%	558	95%	619	97%	567	97%	686	98%	
Outside paediatric oncology centre	115	4%	4	29	6%	31	5%	21	3%	18	3%	16	2%	
Immunophenotype^a^														0.16
BCP-ALL	2562	86%	99	412	87%	502	86%	556	87%	489	84%	603	86%	
T-cell ALL	424	14%	16	64	13%	84	14%	83	13%	95	16%	98	14%	
Unknown (<1% of total)^b^	11		0	5		3		1		1		1		
Down syndrome (only for pts in DCOG registry)														0.31
Yes	77	3%	3	9	2%	16	3%	14	2%	22	4%	16	2%	
No	2805	97%	108	443	98%	542	97%	605	98%	545	96%	670	98%	
Unknown	115			29		31		21		18		16		

*BCP-ALL* B-cell precursor acute lymphoblastic leukaemia.

^a^As confirmed by the DCOG laboratory, if not known in the DCOG registry, the NCR morphology code was taken.

^b^Unknown if not known in the DCOG registry.

^c^6 years period.

Over time patients were increasingly treated at a paediatric oncology centre, 94% in the period 1990–94 compared to 98% in the period 2010–15 (*p* < 0.01) (Table 1). In the last period, 2010–15, only 16 patients were not known in a DCOG centre because of treatment abroad (*n* = 4), treatment at an adult ward (*n* = 9) or death at first presentation at a hospital (*n* = 3).

### Trends in incidence rates

**Fig. 1:**
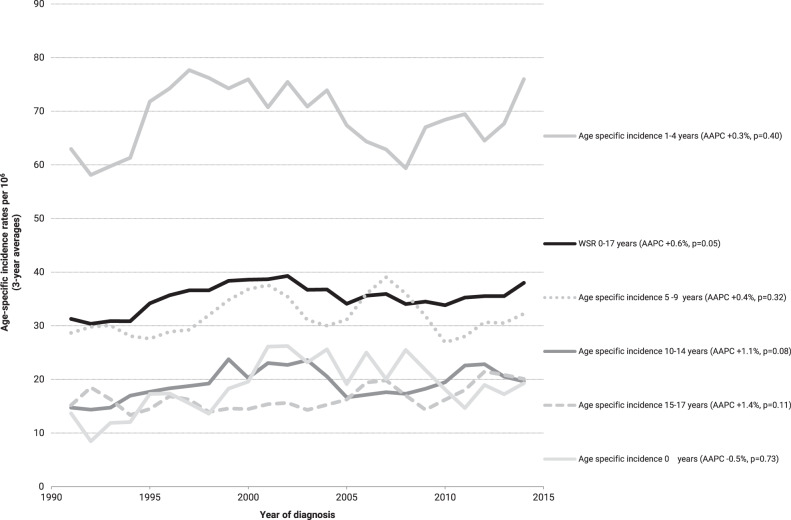
Time trends in incidence of patients aged <18 years with ALL by age groups in the Netherlands, 1990–2015. Three-year moving averages of the age-standardised incidence rate of ALL (standardised according to the World Standard Rate, WSR) and age-specific incidence rates are shown. The average annual percentage change (AAPC) was estimated for each year of diagnosis with linear regression analyses.

On average, 115 patients (range 87–147) were diagnosed with ALL annually. The world standardised incidence rate for patients aged 0–17 years (WSR 0–17) increased over time by 0.6% per year (*p* = 0.05), from 30 per million person-years in 1990–94 to 37 in 2010–15. This increase did not pertain to any age group (Fig. 1) or gender (Supplementary Table S1). BCP-ALL increased over time by 0.6% per year (*p* = 0.06), from 26 per million person-years in 1990–94 to 32 in 2010–15 (Supplementary Table S1). However, for patients aged 10–14 years the increase was significant (AAPC + 1.4%, *p* = 0.04). T-cell ALL only showed an increasing trend for young adolescents (15–17 years) from two patients per year on average in the 1990s to four in 2010–15 (AAPC + 3.7%, *p* = 0.01) (Supplementary Table S1).

### Trends in overall survival

**Fig. 2:**
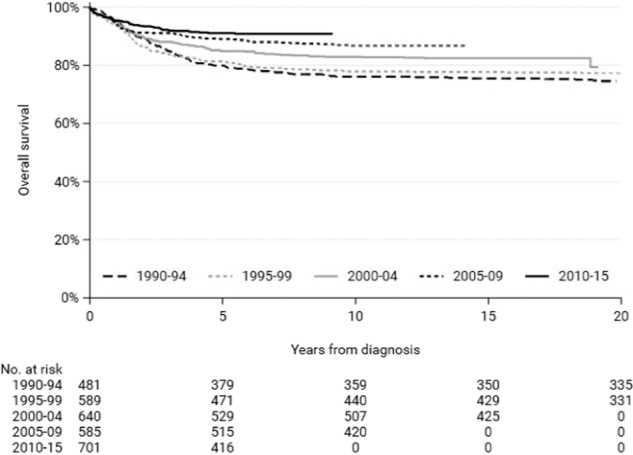
Time trends in overall survival of patients aged <18 years with ALL in the Netherlands, 1990–2015. The 1-year overall survival did not improve over time, *p* = 0.70. The 5- and 10-year overall survival did improve over time, both *p* < 0.01. The *p* for trend was tested with streg and adjusted for follow-up time.

**Fig. 3:**
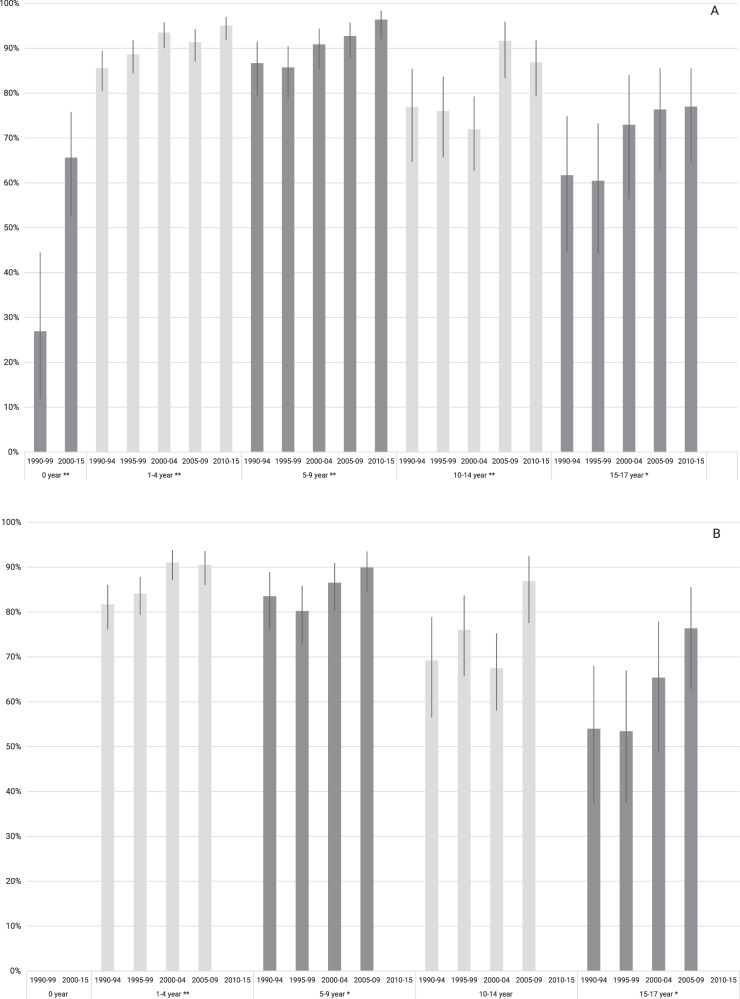
Time trends in overall survival of patients aged <18 years with ALL by age groups in the Netherlands, 1990–2015. Five (**a**) and 10-year (**b**) overall survival with corresponding confidence intervals, corrected for follow-up time. Ten-year overall survival for infants, patients aged <0 years is not given due to <20 patients in this group. And for patients diagnosed in the last period, follow-up time is not sufficient to report 10-year survival. * Indicates significant improvement of survival over time for that age group, *p* ≥ 0.01 and *p* < 0.05 ** Indicates significant improvement of survival over time for that age group, *p* < 0.01 *P* for trend adjusted for follow-up time.

Five-year overall survival increased from 80% (SE 2%) in 1990–94 to 91% (SE 1%) in 2010–15 (*p* < 0.01) (Fig. 2). Ten-year overall survival increased from 76% (SE 2%) in 1990–94 to 87% (SE 1%) in 2005–09 (*p* < 0.01) (Fig. 2). Five-year survival significantly increased for infants aged <1 year from 27% in 1990–99 to 66% in 2000–15 (*p* < 0.01); for patients aged 1–4 years from 86% in 1990–94 to 95% in 2010–15 (*p* < 0.01); for patients aged 5–9 years from 86% in 1990–94 to 96% in 2010–15 (*p* < 0.01); for patients aged 10–14 years from 72% in 1990–94 to 85% in 2010–15 (*p* < 0.01); for patients aged 15–17 years from 57% in 1990–94 to 74% in 2010–15 (*p* = 0.02) (Fig. 3a and Supplementary Table S2). The 10-year overall survival did also increase significantly for all age groups, only non-significantly for patients aged 10–14 years. (Fig. 3b and Supplementary Table S2).

Five-year overall survival significantly increased for both boys and girls; for boys from 75% in 1990–94 to 90% in 2010–15 (*p* < 0.01); for girls from 86% to 91% (*p* = 0.04) (Supplementary Table S2). Ten-year overall survival also significantly increased for boys, from 72% in 1990–94 to 89% in 2005–09 (*p* < 0.01). Five- and 10-year overall survival significantly increased for BCP-ALL, from 81% in 1990–94 to 93% in 2010–15 (*p* < 0.01) and from 77% in 1990–94 to 89% in 2005–09 (*p* < 0.01), respectively. Five and 10-year overall survival for T-cell ALL did not improve (Supplementary Table S2).

### Determinants for risk of death

The multivariable analysis for the risk of dying within 5-years after diagnosis, adjusted for follow-up time, demonstrated a significant decrease in the hazard ratio (HR) during the periods 2005–09 and 2010–15 (HR 0.5, *p* < 0.01 and HR 0.4, *p* < 0.01) compared to 1990–94 (Table 2). Infants, children aged 10–14 years and young adolescents of 15–17 years exhibited an increased risk of death compared with children of 1–4 years at diagnosis (HR 8.2, *p* < 0.01, HR 2.1, *p* < 0.01 and HR 3.5, *p* < 0.01, respectively). Patients with a T-cell ALL were at higher risk of dying compared to patients with BCP-ALL (HR 1.9, *p* < 0.01) (Table 2).

**Table 2:** Multivariable analysis for the risk of dying from acute lymphoblastic leukaemia for patients aged < 18 years in the Netherlands between 1990 and 2015.

	*N*	HR^a^	95% CI	*P* value
Period				
1990–94	481	Ref.		
1995–99	589	0.9	0.7–1.2	0.56
2000–04	640	0.7	0.5–0.9	0.01
2005–09	585	0.5	0.1–0.3	<0.01
2010–15	702	0.4	0.1–0.3	<0.01
Sex				
Male	1744	Ref.		
Female	1253	0.9	0.7–1.1	0.19
Age groups (years)				
0	90	8.2	5.8–12	<0.01
1–4	1385	Ref.		
5–9	796	1	0.8–1.4	0.79
10–14	479	2.1	1.6–2.8	<0.01
15–17	247	3.5	2.6–4.7	<0.01
Immunophenotype				
BCP-ALL	2562	Ref.		
T-cell ALL	424	1.9	1.5–2.4	<0.01
Unknown	11	ND		

*BCP-ALL* B-cell precursor acute lymphoblastic leukaemia, *HR* hazard ratio, *CI* confidence interval, *ND* not done.

^a^In this multivariable analysis, each covariate is simultaneously adjusted for all other covariates and follow-up time. Hazard ratios represent risk of death within 5 years from diagnosis compared to the reference category.

### Site of treatment and trends in overall survival for patients aged 15–17 year

**Fig. 4:**
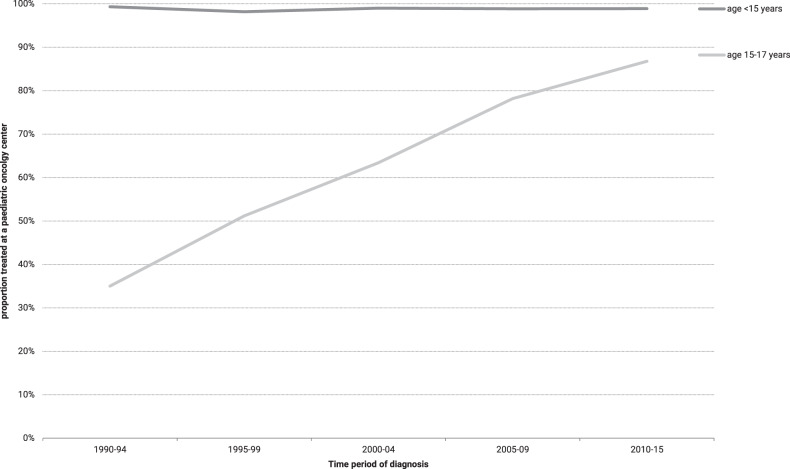
Proportion of patients with ALL treated at a paediatric oncology centre by age groups, 1990–2015. Age groups are displayed in dark gray as age <15 years and light gray age 15–17 years, respectively.

The percentage of patients aged 15–17 year and treated at a paediatric oncology centre increased significantly (*p* < 0.01) over time, being 87% (*n* = 59) during 2010–15 compared with 35% (*n* = 14) during 1990–94  (Fig. 4). To determine whether the site of treatment also affected outcome, we developed two multivariable analyses models. The first demonstrated a decreased risk of death over time for the two most recent periods (2005–09 HR 0.4, *p* = 0.03 and 2010–15 HR 0.5, *p* = 04, respectively). Addition of site of treatment, i.e. adult oncology versus paediatric oncology resulted in the loss of significance for the HRs of the recent periods of diagnosis (HR 0.6, *p* = 0.25 and HR 0.8, *p* = 0.56, respectively). In this second model, site of treatment appeared to be the most discriminative factor for reduced risk of death, i.e. an HR 0.3 for patients treated at a paediatric oncology centre compared to treatment outside a paediatric oncology centre (*p* < 0.01, Table 3)

**Table 3:** Univariate and multivariable analyses for the risk of dying from acute lymphoblastic leukaemia for patients aged 15–17 years in the Netherlands between 1990 and 2015.

		Univariate analysis				Multivariable analysis, 1st model				Multivariable analysis, 2nd model			
	*N*	HR	95% CI		*p* value	HR^a^	95% CI		*p* value	HR^a^	95% CI		*p* value
Period													
1990–94	40	Ref.				Ref.				Ref.			
1995–99	43	0.9	0.5	1.8	0.85	0.9	0.5	1.7	0.73	1.0	0.5	1.9	0.94
2000–04	41	0.6	0.3	1.3	0.23	0.6	0.3	1.3	0.18	0.7	0.3	1.4	0.30
2005–09	54	0.5	0.2	1.0	0.04	0.4	0.2	0.9	0.03	0.6	0.3	1.4	0.25
2010–15	68	0.5	0.3	1.0	0.06	0.5	0.3	1.0	0.04	0.8	0.4	1.7	0.56
Sex													
Male	169	Ref.				Ref.				Ref.			
Female	77	1.2	0.7	1.9	0.48	1.2	0.8	2.0	0.41	1.5	0.9	2.4	0.14
Immunophenotype													
BCP-ALL	179	Ref.				Ref.				Ref.			
T-cell ALL	67	1.5	0.9	2.4	0.12	1.6	1.0	2.6	0.06	1.6	1.0	2.6	0.07
Site of treatment													
Outside paediatric oncology centre	83	Ref.								Ref.			
Paediatric oncology centre	163	0.3	0.2	0.5	<0.01					0.3	0.2	0.5	<0.01

In the first multivariable model we did not consider site of treatment, and this model shows significantly lower risk of death in recent periods of diagnosis compared to the reference period 1990–1994. In the second multivariable model we added site of treatment which results in disappearance of the discriminative effect of period of diagnosis and a significantly lower risk of death for patients treated in a paediatric oncology centre.

*BCP-ALL* B-cell precursor acute lymphoblastic leukaemia, *HR* hazard ratio, *CI* confidence interval.

^a^In the multivariable analysis, each covariate is simultaneously adjusted for all other covariates, and follow-up time. Hazard ratios represent risk of death within 5 years from diagnosis compared to the reference category.

### Trends in mortality rates

Mortality rates below the age of 20 years at time of death decreased remarkably from 9.5 per million children in 1980–84 to 2.8 in 2010–16 (a decline of 4.0% per annum, *p* < 0.01). In the first period on average 40 young people died per year compared to 11 per year in 2010–16 (Supplementary Table S3). Also for the period 1990–2016 the AAPC trend analysis remained significant. Low numbers did not allow to observe a trend in girls below age 5 nor aged 10–14 year at death (Supplementary Table S3).

## Discussion

This is the first population-based study describing trends in incidence, survival and mortality for children and adolescents aged <18 years with ALL in the Netherlands. Over a 26-year period we observed stable incidence rates and increasing survival rates for all ages. The progress made is supported by steadily decreasing, independently assessed, mortality rates for all age groups. Markedly more patients of 15–17 year were treated at a paediatric oncology centre which – in a subgroup analysis – improved their outcome significantly compared with those who were not treated at a paediatric oncology centre.

The age-standardised incidence rate (WSR) of ALL increased with a modest 0.6% per year. For the last period the WSR was 37 cases per million children aged 0–17 years. This incidence rate is similar to other western countries [23, 24], although epidemiologic trend papers report mostly incidence trends for children aged <15 years or including adolescents <20 years. Compared to the reported increase in incidence in the 1990s [4, 5] we can safely assume that incidence remained almost stable after 2000. We were also able to study occurrence of BCP- or T-cell ALL specifically and notice an increase for BCP- ALL in 10–14-year-olds and for T-cell ALL in 15–17-year-olds. Although we did not correct for multiple testing, it is not rare that one or two of the results became positive, due to temporal variation. All in all, substantial influences of environmental factors, either or not pregnancy related, were unlikely to have affected risk of childhood leukaemia in the Netherlands.

Our population-based survival data demonstrated increasing rates over time, with 5-year overall survival of 80% in 1990–94 versus 91% in 2010–15. The population-based study from the CONCORD working group showed similar results for patients aged 0–14 years and year of diagnosis between 1995 and 2009 for north-western European countries comparable with the Netherlands [25]. The COG has reported on the outcome of over 20,000 patients registered in their trials between 1990 and 2005, in which 5-year OS increased from 84% in 1990–94 to 90% in 2000–05 [26] indicating very similar improvements in outcome in both North-America and Europe. Infants, older children and young adolescents had a less favourable prognosis compared to children aged 1–9 years. This might be explained by the higher incidence of unfavourable features such as KMT2A rearrangements [19] in infants and a higher incidence of BCR-ABL like abnormalities [27] and lower incidence of favourable prognostic features such as ETV6-RUNX1 and hyperdiploidy in older patients [12]. The increase in survival rate from 27% in 1990–99 to 66% in 2000–2015 in infants is likely due to the implementation of the Interfant treatment schemes including more intensive use of cytosine arabinoside [18, 19]. It should be mentioned that the confidence intervals for infants are broad due to small numbers. Also, 5-year survival rate of 80% for T-cell ALL in 2010-15 was lower than the 93% for BCP-ALL. Historically, T-ALL patients have had a worse prognosis than other ALL patients [12, 13, 28]. With the better treatment stratifications based on MRD, the outcome for T-ALL patients improved to 81% in 2010–15 but there is still a gap with B-lineage ALL.

Five and 10-year overall survival rates for adolescents aged 15–17 years increased from <60% in 1990–94 to ~75% in 2010–15. The better hospital-based survival rates for adolescents (and young adults) were attained when adolescents were treated on paediatric ALL protocols compared to adult protocols about 15 years ago [29–31]. The percentage of patients aged 15–17 years treated at a paediatric oncology centre increased over time from 35% to 87% in the past 25 years in our study. Interestingly, a multivariable analysis showed that treatment of patients aged 15–17 years in a paediatric oncology centre led to a better outcome. Since the early 2000s young adult ALL treatment protocols have been adapted to the more paediatric like treatment approaches with dose-intensity of non-myelotoxic therapies and stricter timing of subsequent courses [32]. Possibly, there are still differences in management of treatment-related toxicities and/or trial participation in adult versus paediatric centres [33].

In agreement with other studies, mortality rates declined constantly over time at each age group [34, 35]. Increased intensity of induction and reinduction therapy were the first important components of successful ALL treatment protocols at the end of the 1970s and 1980s [36]. We could not report on the incidence and survival in the 1980s because this was before initiation of the NCR. Improvements in chemotherapy and better ways to stratify patients based upon genetic abnormalities and on initial treatment response measured by minimal residual disease [18–20], together with specific protocols for infants and BCR-ABL positive patients, further improved outcome for ALL patients. Supplementary Table 4 shows outcome data of the DCOG protocols used during the time period of the present study. There was no change in death before remission or death in remission over time. The improved survival has been achieved by better initial treatments leading to significantly improved EFS (from 66% to 89%) but part of patients is still rescued by relapse therapy illustrated by the gap between EFS and OS. The rate of stem cell transplantation did not significantly change over time. The proportion of secondary malignancies is below 2% on all DCOG protocols in the time period of the present study [12, 37].

Although detailed information on treatment schemes (initial and relapse treatment), risk group or response status are lacking in the NCR for individual patients, we did not have the intention with this descriptive epidemiological study to study outcome by treatment protocol or risk group. We just wanted to show whether there was progress. Strengths of our study include the linkage with the DCOG clinical registry over the whole study period. We could thus obtain morphology codes of almost all patients by centralised expert haemato-pathology review and determine the proportion of patients treated in a paediatric oncology centre. The latter improvement may be a stimulus for other groups.

All in all, by combining incidence, survival and mortality data we attained a comprehensive picture of the progress against ALL in children and young adolescents in the Netherlands by showing improved survival, especially improved survival of adolescents treated in a paediatric oncology centre, and supported by steadily declining mortality rates. The overall incidence rate was stable, despite two age and type-specific increases.

## Supplementary information

Supplementary figures and tables
