# Supplementary material for: Progress against childhood and adolescent acute lymphoblastic leukaemia in the Netherlands, 1990–2015
Source: Leukemia. 2020 Aug 21;35(4):1001–11. doi: 10.1038/s41375-020-01024-0 (PMC8024196; doi:10.1038/s41375-020-01024-0)
Supplement: Supplementary file 1 — Supplementary figures and tables [file 41375_2020_1024_MOESM1_ESM.docx]

Supplementary figures and tables for the manuscript **Progress against childhood and adolescent acute lymphoblastic leukaemia in the Netherlands, 1990-2015 by Reedijk A.M.J. et al.**

**Supplementary figure 1**

**Linkage between the Dutch Childhood Oncology Group and Netherlands Cancer Registry data**


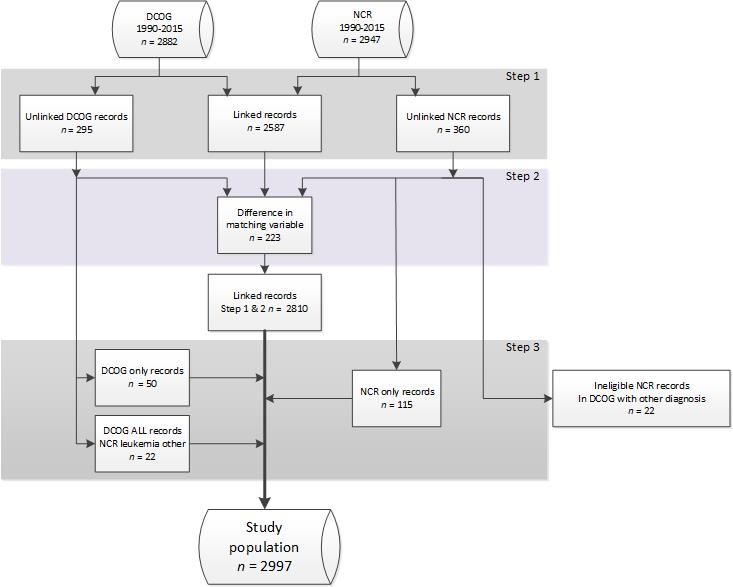


**Legend Supplementary Figure 1**

In order to check for completeness all children below age 18 and diagnosed with acute lymphoblastic leukemia (ALL) between 1 January 1990 and 31 December 2015 were selected from both databases. Linkage between the data files from the Dutch Childhood Oncology Group (DCOG) and Netherlands Cancer Registry (NCR) was performed in three steps. First, data files were merged by date of birth, gender and year of diagnosis which resulted in 2,587 linked records. 223 records had an inconsistency in one of the three merging variables of step 1, but could be added to the linked records. After step 2, 2,810 records were linked. In the third step remaining unlinked records were checked in the other registry by date of birth only. Fifty records were present in the DCOG registry, but could not be identified in the NCR. Another 22 records were also registered in the NCR, but with a different diagnosis; acute myeloid leukemia (AML, *n*=6), non-Hodgkin lymphoma (NHL, *n*=9), mixed phenotype acute leukemia (MPAL, *n*=3) and leukemia not otherwise specified (n=4). With respect to the unlinked NCR records, 115 records were not in the DCOG registry. These patients were included, but additional clinical information and treatment specifics were missing for them. Twenty-two records were registered by the NCR as ALL, but with a different diagnosis by the DCOG; NHL (*n*=18), leukemia not otherwise specified (n=1) and chronic myeloid leukemia (CML, *n*=3). These records were excluded, because the diagnosis by the DCOG was assumed to be most reliable, since they function as a reference laboratory.

After the final step, 2,997 patients could be included in the study.

**Supplementary figure 2 Overview of the DCOG treatment protocols active during the study period**


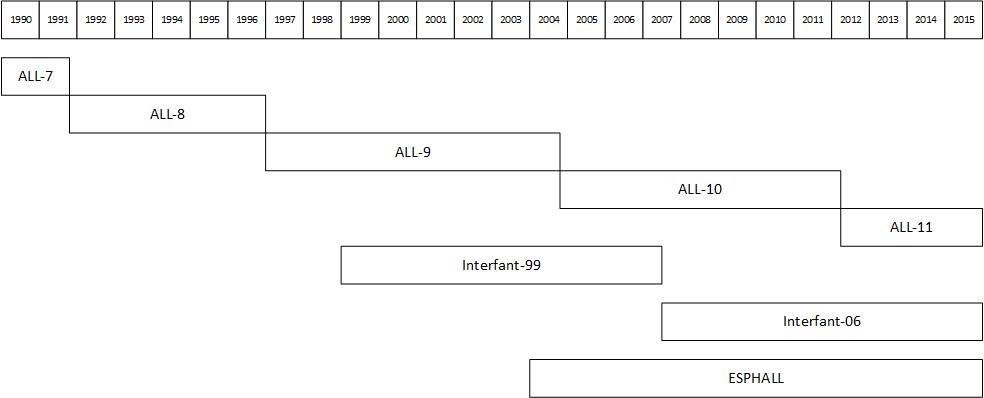


**Footnotes Supplementary Figure 2**

ALL-7: Jul 1988-Sep 1991, 0-15 years of age (total treatment duration was 18 months for all patients)^1^;

ALL-8: Oct 1991-Dec 1996, 0-18 years of age (total duration of chemotherapy for all patients was 24 months)^2^;

ALL-9: Jan 1997-Oct 2004, 0-18 years of age (total treatment duration was 109 weeks)^3^;

ALL-10: Nov 2004-Mar 2012 1-18 years of age (total treatment duration was 24 months)^4^;

ALL-11: Apr 2012-current, 1-18 years of age (2 years of treatment and 3 years for Ikaros positive patients);

Interfant-99:January 1999-January 2006, 0 years of age^5^;

Interfant-06: February 2006-current 0 years of age^6^;

ESPHALL: January 2004-June 2018, irrespective the patient’s age including Philadelphia chromosome-positive acute lymphoblastic leukemia (Ph+ ALL) only^7^.

References

1. Kamps WA, Bokkerink JP, Hahlen K, Hermans J, Riehm H, Gadner H, et al. Intensive treatment of children with acute lymphoblastic leukemia according to ALL-BFM-86 without cranial radiotherapy: results of Dutch Childhood Leukemia Study Group Protocol ALL-7 (1988-1991). Blood 1999 Aug 15; 94(4): 1226-1236.

2. Kamps WA, Bokkerink JP, Hakvoort-Cammel FG, Veerman AJ, Weening RS, van Wering ER, et al. BFM-oriented treatment for children with acute lymphoblastic leukemia without cranial irradiation and treatment reduction for standard risk patients: results of DCLSG protocol ALL-8 (1991-1996). Leukemia 2002 Jun; 16(6): 1099-1111.

3. Veerman AJ, Kamps WA, van den Berg H, van den Berg E, Bokkerink JP, Bruin MC, et al. Dexamethasone-based therapy for childhood acute lymphoblastic leukaemia: results of the prospective Dutch Childhood Oncology Group (DCOG) protocol ALL-9 (1997-2004). Lancet Oncol 2009 Oct; 10(10): 957-966.

4. Pieters R, de Groot-Kruseman H, Van der Velden V, Fiocco M, van den Berg H, de Bont E, et al. Successful Therapy Reduction and Intensification for Childhood Acute Lymphoblastic Leukemia Based on Minimal Residual Disease Monitoring: Study ALL10 From the Dutch Childhood Oncology Group. J Clin Oncol 2016 Aug 1; 34(22): 2591-2601.

5. Pieters R, Schrappe M, De Lorenzo P, Hann I, De Rossi G, Felice M, et al. A treatment protocol for infants younger than 1 year with acute lymphoblastic leukaemia (Interfant-99): an observational study and a multicentre randomised trial. Lancet 2007 Jul 21; 370(9583): 240-50.

6. Pieters R, De Lorenzo P, Ancliffe P, Aversa LA, Brethon B, Biondi A, et al. Outcome of Infants Younger Than 1 Year With Acute Lymphoblastic Leukemia Treated With the Interfant-06 Protocol: Results From an International Phase III Randomized Study. J Clin Oncol 2019 Sep 1; 37(25): 2246-56.

7. Biondi A, Schrappe M, De Lorenzo P, Castor A, Lucchini G, Gandemer V, et al. Imatinib after induction for treatment of children and adolescents with Philadelphia-chromosome-positive acute lymphoblastic leukaemia (EsPhALL): a randomised, open-label, intergroup study. Lancet Oncol 2012 Sep; 13(9): 936-45.

**Supplementary table 1: Incidence for children, aged <18 years and diagnosed with ALL in the Netherlands between 1990 and 2015**

|  |  |  |  |  |  |  | **AAPC** | | | | |
| --- | --- | --- | --- | --- | --- | --- | --- | --- | --- | --- | --- |
| **Incidence Males & Females** | | **1990-94** | **1995-99** | **2000-04** | **2005-09** | **2010-15** | **1990-2015** | **SE** | **95% CI low** | **95% CI high** | **p-value** |
|  | Average number of new cases/ year | 96 | 118 | 128 | 117 | 117 |  |  |  |  |  |
|  | *Age adjusted incidence rate (per 10^6^)* | *30.4* | *36.2* | *37.8* | *34.9* | *36.6* | *0.6* | *0.3* | *0.0* | *1.2* | *0.05* |
| Age (years) | |  |  |  |  |  |  |  |  |  |  |
| 0 | Average number of new cases/ year | 2 | 3 | 5 | 4 | 3 |  |  |  |  |  |
|  | *Incidence rate (per 10^6^)* | *10.2* | *16.5* | *23.7* | *23.7* | *17.0* | *-0.5* | *1.5* | *-3.7* | *2.7* | *0.73* |
| 1-4 | Average number of new cases/ year | 47 | 58 | 59 | 49 | 53 |  |  |  |  |  |
|  | *Incidence rate (per 10^6^)* | *61.7* | *74.6* | *72.9* | *62.9* | *72.7* | *0.3* | *0.4* | *-0.4* | *1.1* | *0.40* |
| 5-9 | Average number of new cases/ year | 26 | 30 | 33 | 36 | 34 |  |  |  |  |  |
|  | *Incidence rate (per 10^6^)* | *28.4* | *30.4* | *33.5* | *35.8* | *30.1* | *0.4* | *0.4* | *-0.4* | *1.3* | *0.32* |
| 10-14 | Average number of new cases/ year | 13 | 18 | 23 | 17 | 21 |  |  |  |  |  |
|  | *Incidence rate (per 10^6^)* | *14.4* | *19.3* | *23.0* | *17.0* | *21.1* | *1.1* | *0.6* | *-0.1* | *2.4* | *0.08* |
| 15-17 | Average number of new cases/ year | 8 | 9 | 8 | 11 | 11 |  |  |  |  |  |
|  | *Incidence rate (per 10^6^)* | *14.2* | *15.6* | *14.3* | *18.3* | *19.0* | *1.4* | *0.9* | *-0.3* | *3.2* | *0.11* |
|  |  |  |  |  |  |  | **AAPC** | | | | |
| **Incidence Males** | | **1990-94** | **1995-99** | **2000-04** | **2005-09** | **2010-15** | **1990-2015** | **SE** | **95% CI low** | **95% CI high** | **p-value** |
|  | Average number of new cases/ year | 53 | 75 | 77 | 65 | 66 |  |  |  |  |  |
|  | *Age adjusted incidence rate (per 10^6^)* | *32.6* | *44.8* | *43.8* | *37.5* | *39.9* | *0.4* | *0.4* | *-0.4* | *1.3* | *0.31* |
| Age (years) | |  |  |  |  |  |  |  |  |  |  |
| *0* | *Average number of new cases/ year* | 1 | 1 | 2 | 2 | 2 |  |  |  |  |  |
|  | *Incidence rate (per 10^6^)* | *8.0* | *12.1* | *21.2* | *21.2* | *20.2* | *1.4* | *1.5* | *-1.8* | *4.6* | *0.36* |
| 1-4 | Average number of new cases/ year | 24 | 38 | 33 | 25 | 27 |  |  |  |  |  |
|  | *Incidence rate (per 10^6^)* | *61.1* | *93.9* | *78.7* | *63.0* | *71.6* | *-0.1* | *0.6* | *-1.3* | *1.1* | *0.87* |
| 5-9 | Average number of new cases/ year | 16 | 18 | 22 | 21 | 19 |  |  |  |  |  |
|  | *Incidence rate (per 10^6^)* | *34.0* | *36.3* | *43.0* | *40.1* | *33.4* | *0.0* | *0.6* | *-1.3* | *1.4* | *0.95* |
| 10-14 | Average number of new cases/ year | 7 | 12 | 15 | 10 | 13 |  |  |  |  |  |
|  | *Incidence rate (per 10^6^)* | *14.7* | *25.0* | *29.2* | *20.5* | *26.1* | *1.8* | *0.9* | *0.0* | *3.6* | *0.05* |
| 15-17 | Average number of new cases/ year | 6 | 6 | 5 | 7 | 8 |  |  |  |  |  |
|  | *Incidence rate (per 10^6^)* | *20.1* | *20.5* | *17.8* | *22.7* | *27.3* | *1.4* | *1.3* | *-1.3* | *4.1* | *0.31* |
|  |  |  |  |  |  |  |  | | | | |

|  |  |  |  |  |  |  |  | | | | |
| --- | --- | --- | --- | --- | --- | --- | --- | --- | --- | --- | --- |
|  |  |  |  |  |  |  |  | | | | |
|  |  |  |  |  |  |  |  | | | | |
|  |  |  |  |  |  |  | **AAPC** | | | | |
| **Incidence Females** | | **1990-94** | **1995-99** | **2000-04** | **2005-09** | **2010-15** | **1990-2015** | **SE** | **95% CI low** | **95% CI high** | **p-value** |
|  | Average number of new cases/ year | 43 | 43 | 51 | 52 | 51 |  |  |  |  |  |
|  | *Age adjusted incidence rate (per 10^6^)* | *28.1* | *27.2* | *31.5* | *32.2* | *33.2* | *0.8* | *0.4* | *-0.1* | *1.7* | *0.08* |
| Age (years) | |  |  |  |  |  |  |  |  |  |  |
| *0* | *Average number of new cases/ year* | 1 | 2 | 3 | 2 | 1 |  |  |  |  |  |
|  | *Incidence rate (per 10^6^)* | *12.6* | *21.1* | *26.3* | *26.4* | *13.5* | *-1.8* | *1.6* | *-5.1* | *1.5* | *0.26* |
| 1-4 | Average number of new cases/ year | 23 | 21 | 26 | 24 | 26 |  |  |  |  |  |
|  | *Incidence rate (per 10^6^)* | *62.3* | *54.4* | *66.9* | *62.8* | *73.9* | *0.8* | *0.6* | *-0.5* | *2.1* | *0.21* |
| 5-9 | Average number of new cases/ year | 10 | 12 | 11 | 15 | 15 |  |  |  |  |  |
|  | *Incidence rate (per 10^6^)* | *22.5* | *24.3* | *23.6* | *31.4* | *26.7* | *1.0* | *0.7* | *-0.5* | *2.5* | *0.18* |
| 10-14 | Average number of new cases/ year | 6 | 6 | 8 | 6 | 8 |  |  |  |  |  |
|  | *Incidence rate (per 10^6^)* | *14.0* | *13.3* | *16.5* | *13.3* | *15.9* | *0.6* | *1.1* | *-1.7* | *3.0* | *0.59* |
| 15-17 | Average number of new cases/ year | 2 | 3 | 3 | 4 | 3 |  |  |  |  |  |
|  | *Incidence rate (per 10^6^)* | *8.1* | *10.4* | *10.7* | *13.6* | *10.3* | *1.4* | *1.3* | *-1.4* | *4.1* | *0.32* |
|  |  |  |  |  |  |  |  |  |  |  |  |
|  |  |  |  |  |  |  | **AAPC** | | | | |
| **Incidence B cell precursor type** | | **1990-94** | **1995-99** | **2000-04** | **2005-09** | **2010-15** | **1990-2015** | **SE** | **95% CI low** | **95% CI high** | **p-value** |
|  | Average number of new cases/ year | 82 | 100 | 111 | 98 | 101 |  |  |  |  |  |
|  | *Age adjusted incidence rate (per 10^6^)* | *26.3* | *31.1* | *33.2* | *29.6* | *31.9* | *0.6* | *0.3* | *0.0* | *1.2* | *0.06* |
| Age (years) | |  |  |  |  |  |  |  |  |  |  |
| *0* | *Average number of new cases/ year* | 2 | 3 | 5 | 4 | 3 |  |  |  |  |  |
|  | *Incidence rate (per 10^6^)* | *10.2* | *13.4* | *23.7* | *23.7* | *16.0* | *-0.5* | *1.7* | *-4.0* | *3.1* | *0.78* |
| 1-4 | Average number of new cases/ year | 44 | 54 | 56 | 45 | 50 |  |  |  |  |  |
|  | *Incidence rate (per 10^6^)* | *57.5* | *69.3* | *69.5* | *58.2* | *68.3* | *0.3* | *0.4* | *-0.5* | *1.1* | *0.40* |
| 5-9 | Average number of new cases/ year | 20 | 24 | 27 | 28 | 24 |  |  |  |  |  |
|  | *Incidence rate (per 10^6^)* | *22.2* | *24.5* | *27.5* | *27.5* | *25.2* | *0.7* | *0.4* | *-0.2* | *1.6* | *0.15* |
| 10-14 | Average number of new cases/ year | 10 | 13 | 17 | 13 | 16 |  |  |  |  |  |
|  | *Incidence rate (per 10^6^)* | *10.6* | *14.3* | *17.1* | *13.0* | *16.3* | ***1.4*** | ***0.6*** | ***0.1*** | ***2.6*** | ***0.04*** |
| 15-17 | Average number of new cases/ year | 6 | 6 | 6 | 8 | 8 |  |  |  |  |  |
|  | *Incidence rate (per 10^6^)* | *11.4* | *11.6* | *10.5* | *13.0* | *12.9* | *0.6* | *1.0* | *-1.4* | *2.7* | *0.52* |
|  |  |  |  |  |  |  |  | | | | |

|  |  |  |  |  |  |  |  | | | | |
| --- | --- | --- | --- | --- | --- | --- | --- | --- | --- | --- | --- |
|  |  |  |  |  |  |  |  | | | | |
|  |  |  |  |  |  |  | **AAPC** | | | | |
| **Incidence T cell type** | | **1990-94** | **1995-99** | **2000-04** | **2005-09** | **2010-15** | **1990-2015** | **SE** | **95% CI low** | **95% CI high** | **p-value** |
|  | Average number of new cases/ year | 13 | 17 | 17 | 19 | 16 |  |  |  |  |  |
|  | *Age adjusted incidence rate (per 10^6^)* | *3.8* | *4.9* | *4.5* | *5.3* | *4.6* | *0.8* | *0.8* | *-0.7* | *2.4* | *0.28* |
| Age (years) | |  |  |  |  |  |  |  |  |  |  |
| *0* | *Average number of new cases/ year* | 0 | <1 | 0 | 0 | <1 |  |  |  |  |  |
|  | *Incidence rate (per 10^6^)* | *0.0* | *2.1* | *0.0* | *0.0* | *0.9* | *NA* |  |  |  |  |
| 1-4 | Average number of new cases/ year | 3 | 4 | 3 | 4 | 3 |  |  |  |  |  |
|  | *Incidence rate (per 10^6^)* | *3.9* | *5.1* | *3.5* | *4.6* | *4.4* | *1.0* | *1.5* | *-2.2* | *4.2* | *0.52* |
| 5-9 | Average number of new cases/ year | 5 | 6 | 6 | 8 | 4 |  |  |  |  |  |
|  | *Incidence rate (per 10^6^)* | *5.9* | *5.9* | *5.8* | *8.4* | *4.8* | *-0.4* | *1.3* | *-2.9* | *2.2* | *0.78* |
| 10-14 | Average number of new cases/ year | 3 | 4 | 6 | 4 | 5 |  |  |  |  |  |
|  | *Incidence rate (per 10^6^)* | *3.1* | *4.8* | *5.8* | *4.0* | *4.8* | *1.4* | *1.3* | *-1.2* | *4.1* | *0.28* |
| 15-17 | Average number of new cases/ year | 2 | 2 | 2 | 3 | 4 |  |  |  |  |  |
|  | *Incidence rate (per 10^6^)* | *2.9* | *4.0* | *3.9* | *5.0* | *6.2* | ***3.7*** | ***1.2*** | ***1.2*** | ***6.2*** | ***0.01*** |

**Supplementary table 2: Trends in 1, 5 and 10-yr overall survival for patients aged <18 years with ALL according to age, gender, subtype between 1990 and 2015**

| ^&^ As confirmed by the DCOG laboratory, if not known in the DCOG registry, the NCR morphology code was taken. BCP-ALL: B-cell precursor acute lymphoblastic leukaemia  ^ unknown if not known in the DCOG registry |
| --- |

* 6yr period

**Supplementary table 3 Mortality for children, aged <20 years at death, dying from ALL in the Netherlands between 1980 and 2016**

**Supplementary table 4 Outcomes of consecutive Dutch Childhood Oncology Group Protocols during the period 1990-2015**

| **Protocol** | **ALL-7** ^1^  Period 1990-91 | **ALL-8** ^2^  Period 1991-97 | **ALL-9** ^3^  Period 1997-2004 | **ALL-10** ^4^  Period 2004-2012 |
| --- | --- | --- | --- | --- |
| % death - before remission  - in remission | 2%  3% | 1%  2% | 1%  3% | 2%  3% |
| % relapse  5-year EFS % (SE) ^$^  5-year OS % (SE) ^$^  5-year CIR % (SE) ^$^ | 32%  66 (3)  80 (3)  30 (3) | 25%  75 (2)  85 (2)  22 (2) | 15%  83 (1)  88 (1)  13 (1) | 9%  89 (1)  94 (1)  8 (1) |
| % alloSCT - in first CR | ~3% | 2% | 2% | 6% |
| % sec malignancy | 2% | 1% | 0.1% | 1% |

Protocol ALL-11 is still ongoing, this data cannot be presented yet.

^$^ Excluding children older than 15 years, patients with DS, infants and Philadelphia chromosome-positive ALL because of differences in inclusion, adapted from Pieters et al. JCO 2016^4^, table 3.

Abbreviations: alloSCT, allogenic hematopoietic stem cell transplantation, CIR, cumulative incidence of relapse, CR, complete remission, DS, Down syndrome, EFS, event free survival, OS, overall survival, SE, standard error

References

1. Kamps WA, Bokkerink JP, Hahlen K, Hermans J, Riehm H, Gadner H, et al. Intensive treatment of children with acute lymphoblastic leukemia according to ALL-BFM-86 without cranial radiotherapy: results of Dutch Childhood Leukemia Study Group Protocol ALL-7 (1988-1991). Blood 1999 Aug 15; 94(4): 1226-1236.

2. Kamps WA, Bokkerink JP, Hakvoort-Cammel FG, Veerman AJ, Weening RS, van Wering ER, et al. BFM-oriented treatment for children with acute lymphoblastic leukemia without cranial irradiation and treatment reduction for standard risk patients: results of DCLSG protocol ALL-8 (1991-1996). Leukemia 2002 Jun; 16(6): 1099-1111.

3. Veerman AJ, Kamps WA, van den Berg H, van den Berg E, Bokkerink JP, Bruin MC, et al. Dexamethasone-based therapy for childhood acute lymphoblastic leukaemia: results of the prospective Dutch Childhood Oncology Group (DCOG) protocol ALL-9 (1997-2004). Lancet Oncol 2009 Oct; 10(10): 957-966.

4. Pieters R, de Groot-Kruseman H, Van der Velden V, Fiocco M, van den Berg H, de Bont E, et al. Successful Therapy Reduction and Intensification for Childhood Acute Lymphoblastic Leukemia Based on Minimal Residual Disease Monitoring: Study ALL10 From the Dutch Childhood Oncology Group. J Clin Oncol 2016 Aug 1; 34(22): 2591-2601.
